# A review of Indian psychiatry research and ethics

**DOI:** 10.4103/0019-5545.69257

**Published:** 2010-01

**Authors:** A. K. Agarwal

**Affiliations:** Retd. Professor, Department of Psychiatry, K.G’s Medical College, Lucknow, India

**Keywords:** Ethics, Psychiatry, India

## Abstract

Ethics does not seem to be a favorite topic of Indian authors. Electronic search of the IJP web site could only identify six articles which were directly related to ethics. One article discussed the relationship of ethics religion and psychiatry. Another editorial discussed the concept of responsibility in psychiatrists. Other editorial discussed the truth about ‘truth serum’ in legal investigations. One article discussed the ethical aspects of published research. There were two articles that specifically discussed ethical aspects. This write-up provides some details about the ethical aspects of psychiatric practice, specific to India, and emphasizes the need to rediscover ethics in India.

## INTRODUCTION

Ethics do not appear to be a favorite subject for Indian authors. Repeated search by electronics means at the IPS Journal website could only select six articles which are basically related to ethics in psychiatry. Many other articles were also picked up, which had hardly any connection with ethics. A brief review of these articles is essential before we can proceed further.

First article[1] selected is entitled *Psychiatry, Religion and Ethics* from IJP 1965. This is a very interesting article. I am fortunate to know the author who was doing course in Clinical psychology at the same time when I was studying psychiatry at the All India Institute of Mental Health, Bangalore. He was a very considerate individual very helpful and respectful for all. He could stand against authority if he felt he was on the right side. These are the qualities of an ethical person who can risk personal gains to preserve ethical values. This article is basically a reflection of his personality as well as psychiatric teaching of that time. Any mental illness was considered a consequence of psychosocial forces; the biological factors were acknowledged but not emphasized. The hypothesis put forward by Singh in this paper are as follows:

All religions are basically same and all of them emphasize on ethical behavior. Mental illnesses are caused by psychosocial stressors and if one could follow ethical path or the path of righteousness, one would not have any stress and ultimately no mental illness. The mentally ill could also gain health following a similar path. The emphasis is on ethical behavior; even today, if one follows the path of righteousness one would at least be more comfortable, if not fully healthy.

The other two articles are editorials by N. G. Desai[2] and AK Kala,[3] both very important and worth discussion in some detail.

*Responsibility of psychiatrists: Need for pragmatic idealism* is the title of Desai’s editorial. The author quotes Chodoff to enumerate the responsibilities of psychiatrists.

The responsibilities cited are:

- Competence or the need to master their task;
- Ethical behavior or to police their ranks;
- Accountability or to be accountable to public;
- Advocacy or to be advocate for mentally ill persons.

These responsibilities are generally not emphasized. Most clinicians feel comfortable following the traditional clinical role of one-to-one relationship. The emphasis on advocacy and accountability are new concepts which are important in today’s scenario. Desai emphasizes that the responsibility of being a competent psychiatrist with adequate skills seems to be undermined by varying standards of education and indifferent monitoring mechanisms. The training of psychiatrists in this country varies widely from center to center. Poor competence is also ascribed to the perception that psychiatry is not a very exact science and many people feel that exacting skill training is not required. The author further emphasizes that a psychiatrist should be well aware of the different laws that effect mentally ill persons and should respect those laws. Ethical responsibility is often ignored as the ethical guidelines are not clear and effective. The author reiterates that the psychiatrist should be able to accept and identify mental health issue within themselves and their close relatives. He feels that the public expects a mental health professional to be free of mental ill health. The editorials bring in a very relevant concept of concentric responsibilities; must do (obligatory), should do (desirable) and can do (optional) level of responsibilities. One will have to recognize that there would always be wide variations amongst mental health professional but some qualities are must and should be present in each one, while others are desirable, yet some others would add to their effectiveness.

This is an important editorial which emphasizes newer dimensions to ethical behavior like advocacy social responsibility and certain unconventional areas are included.

Kala[3] writes on “ *ethically compromising positions and blatant lies about truth serum*’.

This editorial is very timely as Indian courts and investigating agencies are getting more and more involved with pseudoscientific methods of investigations. It is a well known fact that information obtained during chemical stimulation could be totally unreliable. The evidence thus obtained is not admissible in the court of law. Why are the investigating agencies pursuing these methods more frequently in this country? This appears to be the replacement of third degree methods used earlier. These methods are serious blow to the human rights of the accused. The mental health professionals who participate in such activities are being unethical. This also brings the profession into disrepute as the general public perceives that the psychiatrists must be using similar methods in their day to day practice. This is a timely reminder of potential ethical violation.

Chaturvedi and Somashekar[4] 2009 studied *ethical aspects of published research*. The authors studied the consent procedures and ethical review board’s approval in published research in IJP in 2000, 2003 to 2007. Results indicated that informed consent was mentioned in 51% studies in 2000, which gradually rose to 82% by 2007. Ethics committee approval was mentioned in 2% in year 2000 and increased to 25% by 2007. Written consent procedures were used in only 40%. This is a very important area which has not aroused enough interest. There should be a very thorough screening of all research articles before publication in our journal. The purpose of such screening should not be negative to inhibit research but should be positive to promote ethical research. IJP should have an ethical consultancy group where researchers could be able to get ethical guidance at the time of planning the research.

Srinivasamurthi *et al*.[5] surveyed the medical profession on ethical issues. This article was not published in IJP the survey revealed that only 23% of mailed questionnaires were returned. The approximate return rate has been found to be the same in most such studies. What was more important in this survey was - majority of respondents were involved in research while very few clinicians responded. This may indicate that researchers were more concerned with ethics than the clinicians. 93% of the respondent felt that ethical training should be started at undergraduate level. This author has published two articles on ethics in psychiatry in IJP[6,7] and some at other places.[8–10]

Ethics does not appear to be a favorite topic of Indian psychiatrists and mental health workers. However, the story of our progress on ethical issues is worth recall. There has always been an ethical committee in the Indian Psychiatric Society but there were no specific input by this committee. This author was appointed the chair person of ethical committee in 1986. The author along with some other friends decided to hold a workshop on Ethics. This led to the issue of funding as this was a workshop on ethics so funding from pharmaceutical industries was avoided. It was decided to approach University Grant commission for fund allocation. When we approached the authorities of UGC they told this author point blank that they only finance project of institutions which are under Universities but the K.G’s Medical College at that time was under U.P. Government so funding was not possible. As we were in the process of leaving his office, the officer concerned asked as to what was the topic of the workshop. When they were informed that it is a workshop on Ethics, he suddenly showed interest and funded the whole workshop including the printing of the proceedings. This incident is given in some detail to emphasize that ethics still produces positive vibes in many and ethical behavior is still in demand. The proceedings of this workshop were published in1989.[9] Deliberation led to the formulation of ethical guidelines of IPS which were adopted by the society in Cuttack conference. These guide lines were never discussed again and there had not been any discussion on ethics in any of the forums of the Indian Psychiatric Society. There had been one or two symposiums on ethics of drug trials. Medical council of India[11] has published ethical rules in 2002.These are guidelines but they have the authority of law. One has to remember that every doctor in India has to follow them; there could be additional specialty specific guide lines. American psychiatric association also first details ethical guidelines of American medical association than elaborates on guidelines specific to psychiatry. There are some other publications related to this topic. The following is based on these publications as well as on the ethical guidelines of American Psychiatric Association.

## ETHICAL ISSUES SPECIFIC TO PSYCHIATRY

### Diagnosis

The diagnostic method used in psychiatry is behavioral observations and there are hardly any investigations that can clinch the diagnosis. Symptoms used in diagnosis can be seen normally; there is only a difference in frequency or severity. Deviance from social norms was at times considered a symptom of psychiatric illness. Though the reality of mental illness is now accepted by all there are still gray areas which do not fulfill the robust criteria of diagnosis e.g. personality disorders or grief. One will have to be very careful in diagnosing a person mentally ill and continuous revision and updating of diagnostic criteria is the only way by which we can avoid the criticism of being a pseudoscience. It is the duty of the mental health professional to note ones observations correctly, get inputs from sources like family, employer, and primary physician and then draw conclusion. A scientific diagnosis is essential to ethical practice. Psychiatry has been misused in many countries where the non conformists were sent to mental hospitals to crush dissent.

#### Psychiatrist patient relationship

All medical treatment is based on physician-patient relationship. This relationship is generally between two equally autonomous people who can evaluate the advantages/disadvantages of the relationship and may decide to continue or break the relationship when they want. However, the situation could be quite different in the mentally ill. The patient, because of his illness which affects his thinking, emotions and cognitive functions may be less empowered than the psychiatrist and may not be able to take independent decision. This inequality puts greater burden on the psychiatrist and he must ensure that the patients well being, should be foremost concern and therapist’s personal benefits should be kept in the background. Patient should not be exploited physically, sexually, emotionally or financially.

Patient-doctor relationship in the field of mental health is prolonged and as intimate personal details are being disclosed, there is a likelihood of development of strong relationship (transference).These relationships may lead to inappropriate behavior of strong love or hatred and the therapist needs to be vigilant and not let these vitiate the therapeutic relationship.

#### Involuntary treatment

Most psychiatric patients believe they are not ill and refuse treatment. Hence they are treated against their wishes. Laws of each country recognize this problem and therefore they have provided legal safeguards for there treatment. Mental Health Act 1987 of India[12] provides for involuntary hospitalization with the consent of caring relative or through legal procedure. Unfortunately or fortunately, majority of the mentally ill are treated either as out-patients or in psychiatric units of general hospitals. These patients are either treated with relative’s consent or they are made to sign a consent which they do not understand. Szasz calls this practice of voluntary admission an unacknowledged example of medical fraud.[7] The current situation in India can lead to unethical practice where a person’s autonomy is restricted without legal safeguards.

Madrid declaration states that “Involuntary intervention is a great infringement of the human rights and the fundamental freedom of a patient. Therefore, specific and carefully defined criteria and safeguards are needed for such intervention. Hospitalization or treatment against the will of the patient should not be carried out, unless the patient suffers from severe mental illness. Involuntary intervention must be carried out in accordance with least restrictive principle.”

Hawaii declaration of WPA states, “No procedure must be performed or treatment given against or independent of patient’s will, unless the patient lacks capacity to express his or her own wishes, or owing to psychiatric illness can not see what is in his best interest or, for the same reason is a severe threat to others. In these cases, compulsory treatment may or should be given, provided it is done in the patient’s best interest and over a reasonable period of time, a retroactive informed consent can be presumed and whenever possible, consent has been obtained from someone close to the patient. As soon as the above conditions for compulsory treatment or detention no longer apply, the patient must be released, unless he or she voluntarily consents to further treatment. Whenever there is compulsory treatment, there must be an independent and neutral body of appeal for regular enquiry into these cases. Every patient must be informed of its existence and permitted to appeal to it, personally or through a representative without interference by hospital staff or by anyone else”.

The major situations where one could recommend involuntary hospitalization, according to Mental Health Act 1987, are as follows:

1. Patient is dangerous to self/others
2. There is a possibility of improvement by hospitalization
3. The patient is incompetent

The psychiatrist and the mental health team should closely monitor the patient for competence to make informed decisions and as soon as the patient is able to make such decisions he should be given the liberty of choosing the treatment option. This aspect is not really practiced in this country. Large numbers of mentally ill patients who have improved are still in the hospitals as there family members are not ready to accept them or they have no place to go or the family wants them to stay in hospital to avoid the inconvenience of keeping them at home. This is a serious lapse of patient’s human rights and the profession need to look into it.

Most of the patients in this country are being treated as outpatients. The uncooperative patient is given medicine surreptitiously or by injections against their will with the consent of family members. This is being done keeping in mind the principle of beneficence and is ethically acceptable. The problem with this practice is that it can be misused by some. Secondly, this has resulted in revolving door phenomenon. The relatives give the medicine to the patient only till such times the acute symptoms subside and thereafter stop it. Some times the patient refuses to take it but often there is a mutual hidden agreement between the patient and the relatives that the medicines are no longer required. This leads to frequent relapses. There is an urgent need to develop patient and family information material, which could help them decide when to continue or stop treatment.

#### Confidentiality

It refers to therapists responsibility of not disclosing information learned during treatment to any one without the patient’s permission. It is the patient’s privilege and should be respected except in certain predefined situations. It is important to differentiate between the patient’s privilege of respect to his confidentiality and the legal requirements of withholding medical information. These two are not the same. It is prudent to inform the patient that the information provided by him will be kept confidential except in the exceptions as enumerated later. Earlier the families were not provided the confidential information but now the families are considered an important part of therapeutic activity and the patient’s need to be encouraged to share required information with the family. Therapists who do not take care of the confidence of people by their acts of omission or commission would be considered unethical. Doctors who expose patient’s private part in front of others are guilty of breach of ethics. Psychiatrists who ask confidential matter from the patients in front of relatives or other strangers are guilty of the same. The case records of patients should not lie unguarded where they could be accessed by any one. It would show that the psychiatrist is not taking enough precautions in protecting the patient’s confidences. If case records are being maintained in the electronic form one need to be more vigilant so that these could not be accessed by unauthorized persons. It is always prudent not to mention seriously incriminating information in the case sheet where it could be accessed by others. Gossiping about patients or publishing case records without hiding the identity of the patient or without his permission would amount to breach of confidence.

With whom one can share this information without the permission of the patient.

The confidential information could be shared with the treating team which may include nurse, psychologist, social workers and other personnel actively involved in treatment. All of them should be sworn to keep the confidentiality and in case of any breach by anyone of them, the responsibility would of the psychiatrist. Very often the family members, the employer and the authorities wish to know about the patient. The psychiatrist should provide confidential information only after obtaining explicit permission from the patient.

Major exceptions to confidentiality

1. Patient consents to release information to family members, employer, and insurance company or to anybody else.
2. Tarasoff duty: When patient’s acts are likely to harm others then it is the doctor’s responsibility to protect others from harm. If a patient informs the doctor that he is going to kill some one than it is doctors ethical responsibility to take appropriate action so that the intended victim could be protected. We often come across instances when a mentally impaired person can harm others by his negligent behavior e.g. Persons engaged in running public transport like buses trains etc, officers in police army or doctors. It is the responsibility of the treating doctor to take appropriate action and avoid the possibility of public harm.
3. Emergencies: When patients life is at stake the confidences can breached so that he gets efficient care.
4. Mandatory reporting: Whenever any kind of human right violation is observed like child abuse, female abuse than it has to be reported to appropriate authorities. Persons who are HIV positive and plan to marry should be encouraged to inform the spouse. If they do not agree to do so then the psychiatrist can inform the spouse to protect him or her.
5. Court orders: If a court of law asks for some confidential information than psychiatrist should seek permission from the patient. If the patient refuses permission, the psychiatrist should inform the court of the same. If the court even then directs that he should reveal the confidences than he could do so.
6. Patient initiates litigation against the psychiatrist: The psychiatrist can reveal such confidences that are directly relevant to the case.

Principles to follow when breaching confidence is necessary

1. Inform the patient wherever possible. Seek his consent
2. Disclose only the relevant information
3. Document rationale for action

Confidentiality endures after death, and one should not disclose information unless next of kin provide consent.

#### Honesty and trust worthiness

Honesty and trust are the basis of doctor patient relationship. Honesty entails positive duty to tell the truth as well as the negative duty not to lie or intentionally mislead some one. Psychiatrist often learns many a sensitive information about the patient and some times try to hide the information to protect the patient. In general, omission (intentional failure to disclose) and evasion (avoidance of telling the truth) will undermine a constructive relationship and is not appropriate. Large number of patients and their relatives repetitively request the psychiatrist to provide a certificate of illness that may not affect their jobs or marriages. Acceding to such requests would undermine the principles of honesty and trustworthiness and would bring down the prestige of the profession.

#### Non participation in fraud

Fraud is an action that is intended to deceive and ordinarily arises in the context of behavior that seeks to secure unfair or unlawful gains. This is an extension of the principle of honesty and trustworthiness. One may be tempted to help a patient by providing false information regarding his illness or treatment but in the long run it is going to undermine the trust of the patient as well as others especially third party providers. The specific examples are writing the prescription in some other name where patient could get free medicines, or changing the diagnosis in the certificate or not informing the employer of the potential hazards this illness may entail on the work of the patient. The psychiatrist should avoid all kind of wrong doings to uphold the dignity of the profession. Such conduct will be beneficial to the patient in long term.

#### Informed consent

The concept of informed consent has gain importance since 1950. The concept is still evolving. This concept is centered on three aspects;

Information - What is to be informed and how.

Is the patient competent to understand the information and can make rational decisions.

Can the patient take autonomous decisions without being influenced by the disease process cultural factors, or other extraneous factors?

Information - The patient should be informed about the nature of the illness. What is the diagnosis and what is the likely course if untreated and what is likely to happen with different treatments available. What treatment the doctor is recommending and why. What are the likely side effects of the treatment? What is the duration of the treatment? What is the cost of the treatment? What is the most cost effective treatment? Whether this treatment has strong evidence base.

Second question to be considered is how much is to be informed. This varies from State to State in USA. Some state requires that what is generally informed by doctors should be informed. While others would require reasonable information which an individual would require for mature decision making. The second option appears more reasonable. This issue has not been discussed in this country. If one is informing about the side-effects of a treatment should he inform only the common and less harmful side effects or he should inform rare but fatal side effects. The later could scare the patient.

The third question is how to inform. Should the information be oral or written? In research written information is mandatory. Oral information can be distorted and the patients and relatives may be induced to accept what the doctor’s desire. Ideal should be written information which could be further discussed. The next question is that who should obtain consent, the treating clinician or some other person who may not have any stakes in treatment.

Competence - Can the patient or the relative comprehend what is being informed. Can he process this material to reach a sound decision? Ordinarily, every adult is considered competent and should be able to make a choice. The psychiatrist should assess the ability of the patient to comprehend information to process it rationally, and to reach sound decisions. Sometimes patients who have cognitive deficits or psychotic symptoms can give a valid consent, if information is presented in small parts. In reality not even many mentally healthy person can understand the medical jargon and not many can decide whether to consent for ECT or drugs. Such decision making is mostly influenced by hearsay, prejudices and stigma. Ideally, a medical decision could only be made by a medical man. The medical men should discuss the decision with the patient and family members and they should be informed of all the advantages and the disadvantages of the proposed course of action. The patient could also be encouraged to seek a second opinion from a competent psychiatrist of their choice.

Autonomy - Can the patient make an autonomous decision. Often the illness can affect the individual in such a way that he may be incapable to make an autonomous decision. Depressed patient may be so fed up with life that he may consent to any treatment without fair consideration. Persons suffering from alexithymia will not be able to make a decision. The situation in India is more complex due to deficient manpower. There are very few mental health facilities and thus the patient has to accept what is being offered as there are no alternatives. Poverty could be another factor.

The concept of informed consent is surrounded by controversies. Some feel it is a consequence of defensive medical practice in the US, where litigations are frequent. When consent has been obtained than the legal liability of the clinician is very much diminished. Large number of psychiatric patient does not consider them to be ill and thus refuse consent. This will results in delayed or no treatment. Autonomy of the patient can also be restricted by biases and prejudices. Many patients believe in some the following.

- Mental illnesses are a result of ones own faults and they should be controlled by them personally.
- Mental illnesses are caused by supernatural powers hence the religious treatment is appropriate.
- Mental illnesses are caused by circumstances and unless they are changed no treatment can be helpful.
- Psychiatrist prescribes sleeping pills and that leads to addiction and not treatment.
- Psychiatric treatments like ECT may cause more harm than the illness.
- If one takes psychiatric treatment once then he has to take it for the life time.

These and similar biases and prejudices influence the patients decision making process. Therefore it is essential that the clinician educate and inform the patients about the reality of these myths.

The consent should not be blanket consent as is often practiced in this country. There is a consent form on the first page of the case record and each patient or the relative is asked to sign this blanket consent. Consent should be obtained for each procedure separately and the patient can give a limited consent or may revise it after some time. The procedure of consent has not been sufficiently discussed in this country and it is high time that we evolve consent procedures that are relevant to our needs.

The Supreme Court of India[13] in its judgment on January 16, 2008, held that a doctor has to seek and secure the consent of the patient before commencing a ‘treatment’. Giving the judgment the three judge bench said that “the consent so obtained should be real and valid; the patient should have the capacity and competence to consent; his consent should be voluntary; and his consent should be on the basis of adequate information concerning the nature of the treatment procedure, so that he knows what he is consenting to” This judgment comprehensively sums up the consent process and the Supreme Court decisions have the power of the law.

#### Exceptions to informed consent

1. Emergencies
2. Therapeutic privilege-In situations where giving of all the information necessary to make a decision could harm the patient the therapist can withhold information. This should be a rare exception. Under such circumstances the rationale of withholding information should be recorded.
3. Incompetence due to mental condition or in cases of minors
4. Waiver-when the patient has explicitly stated that he accepts all the decisions of the treating team

#### Professional competence

The professional competence of the doctor is the key to ethical practice. A person who is not sufficiently trained or has not kept himself up to date in knowledge can not provide competent care. The psychiatrist should continue to update his knowledge. Medical Council guidelines recommend that the professional should join professional societies where he could update knowledge. It is time that we should evolve a more concrete method of lifelong learning and its evaluation.

Every clinician should be aware of his expertise and should not treat conditions falling outside his expertise. A cardiologist treating a psychiatric patient or vice versa are clear examples of unethical behavior.

One can treat beyond his expertise only in emergencies or when specifically trained professional is not available. Physicians who are incompetent due to age or illness should stop clinical work. It is the duty of every physician to report such persons to appropriate authority so that they do not harm the unsuspecting patient.

Physicians involved in unethical behavior should also be identified and reported so that the profession could not be painted black due to nefarious activities of few. Unfortunately we Indians have a great capacity to tolerate deviance due to which black sheep of the profession thrive.

#### Relationship with colleagues

The psychiatrist should treat their colleagues with respect. The opinion of a colleague should not be criticized in front of the patient and his relatives. If the colleague is involved in unethical behavior than the same could be reported to appropriate authorities.

#### Referral

If one finds that he does not have enough expertise to treat a particular patient then he could be referred to a person who has the requisite skills. If a patient has not responded reasonably to treatment, the patient should be referred to a colleague for second opinion or further treatment. If an unconventional treatment is proposed than a second opinion should be sought. If the patient is not satisfied with the treatment than he should be encouraged to seek a second opinion.

#### Charging of fees from colleagues and their families

The ancient medical ethics prohibited the charging of consultation fee from colleagues, teachers and their families. This issue needs to be debated keeping the current realities in mind.

#### Boundary violations

A boundary may be defined as the “edge” of appropriate professional behavior, transgression of which involves breaching the clinical role. Boundary issues involve the therapist’s role and his relationship with the patient and his family. Issues may include the following

- Time and place of consultations, contacts on phone, in social meetings etc
- Accepting or giving of gifts, money
- Making inappropriate arrangements of payment of dues in cash, kind or barter system
- Types of clothes the doctor wears or the language he uses could involve boundary violations
- Talking about self in therapeutic situation
- Physical contact including sex but not limited to it

The therapeutic relationship between a doctor and the patient is established solely with the purpose of therapy and whenever this relationship deviates from its basic goal of treatment it is called boundary violation and becomes non therapeutic. In psychiatry, as the therapeutic relationship is prolonged and more personal as many confidential matters are discussed, there is likelihood of developing strong emotional bonds. This may lead to non therapeutic activity.

1. Sexual activity with a patient, ex-patient or with the patient’s family member is unethical.
2. Business relationship with a current patient is unethical except when one is living in a small community where such relationship can not be avoided.
3. Ideological: Any clinical decision should be based on what is best for the patient; the psychiatrist’s ideology should play as little a part as possible in such decisions. The psychiatrist may be against inter-religion marriages but if the patient wants it than his wishes should be respected.
4. Social: Whenever the psychiatrist and the patient start becoming friendly then the therapeutic relationship is compromised. The objectivity is compromised and factors outside the therapeutic relationship may become destructive to the therapeutic process. Psychiatrist should avoid non therapeutic contact with the patient. Similarly, one should not treat one’s friends. The place of consultation should be clinic and the time should also be during the consultation hours. Any deviations would distort the therapeutic relationship.
5. Financial: If the patient is being treated in a state hospital where no fee is charged then the doctor should not accept any gift in form of cash or goods. Small gifts on special occasions could be justified. Very often, influential people like politicians and government officials may offer special privileges for the doctor or his department but all such concessions or allurements are also unethical. If the psychiatrist charges fees he should keep charges that are reasonable for that area. Still there could be patients who would not be able to pay. There should be a transparent policy of fee reduction. It should not be done differently for different people because that may again distort relationships.
6. Dress and language: Doctor should be dressed formally. Dresses that are flashy or reveal body part in a provocative manner should be avoided. The language used should be formal and abusive or double meaning words should be avoided.

The doctor is responsible for preserving the boundary and he should ensure that boundary violations do not occur. If even a minor violation occurs it is better to transfer the patient to a colleague. The boundary violation typically starts small and become incrementally problematic and the dyad starts sliding down the slope. This is known as *Slippery Slope Concept*.

#### Double agentry

This type of conflict arises when the psychiatrist has responsibility to another agency which might be his employer in addition to his responsibility to the patient. A psychiatrist in the armed forces is duty bound to inform his superiors if any one of his patient develops a psychiatric illness which might be a security risk. Similarly, psychiatrists employed in other industrial organizations could be duty bound to provide certain information to there employers. In western countries where health care is provided by third parties treating doctors are expected to provide information to them.

The psychiatrist should inform the patients at the start of the treatment about his obligations to provide information to specific agencies and an informed Consent should be obtained.

#### Conflict of interest

Conflict of interest is “a set of conditions in which professional judgment concerning a primary interest (such as patients’ welfare or validity of research) tends to be unduly influenced by a secondary interest (such as financial gain)”. The relationship between physician and pharmaceutical industry, research on patients and physicians relationship with health providers are examples of such conflict of interest.

Relationship with pharmaceutical industry is the center of focus these days. It is said that the pharmaceutical lobby influence medical publishing, as well as doctors prescribing habits. There is good evidence that pharmaceutical industry is a main sponsor of research. It is believed that nearly 50% of articles dealing with therapeutics appearing in Lancet and BMJ are ghost written.[14] The recent example of inappropriate promotion of drugs like Alteplase and Xigris are testimony of pharmaceutical industries influence on research and publication.[15] Clinician has to be very vigilant that his decision may not be influenced by such misreporting. There is some evidence that even meta- analysis could also be tampered with by interested parties. It is therefore important that the profession remains vigilant It is now accepted by almost all ethical organizations including MCI that only small gifts could be accepted by the doctors from the pharmaceutical industry. Pharmaceutical sponsored Continuing Medical Education (CME) would generally serve the industries interests. Most of our conferences and other research needs are being sponsored by the industry - Is it a healthy sign. This may result in inappropriate prescription of drugs which is totally unethical.

The health service providers also influence clinical decisions. Health authorities in our country insist that the doctors should prescribe only those medicines that are available in the hospital. This interferes with one’s clinical decision making. Similarly in the west where insurance companies fund health care they decide when the patient should be admitted or when he should have psychotherapy thus effecting clinical decision making.

### Ethical issues in day- to-day clinical work

Ethical behavior should be part and parcel of day to day clinical activity. How much information has to be obtained for effective diagnosis and clinical care is an ethical issue which is often ignored. If a patient has a small injury in his toe we need not disrobe him completely. Similarly psychiatric history taking and examination should be need based and unnecessary details of intensely personal matters like sex or other emotional relationship should only be obtained when needed.

Patient should be informed about diagnosis, treatment and likely prognosis.

How much is to be informed and to whom should follow well established practices.

Patient and families will ask many questions and these should be answered. The answer should not be given without proper thought and should be based on available evidence. Usually the relatives of seriously sick patients may ask questions regarding marriage, likelihood of inheriting the disease or the effect of disease on work or family life. All these questions have to be answered after weighing available evidence. If one is not sure of the answer then one can even tell the family members that he will need to find out from some one else. Often the doctors give false assurances or try to hide serious long term consequences to reduce the pain and suffering of the patients, but such acts often misfire. The father of a girl with schizophrenia told this author that if the earlier psychiatrist would have told him that this illness is likely to relapse he would not have married his daughter. A well intended act has led to an emotional disaster. The doctors should be very careful in use of words and should always stick to the truth; well intentioned half lies or evasions are not ethical.

Ethical consideration should influence even minor clinical decisions. Writing costly medicines when same medicine is available at a cheaper cost is an ethical transgression. Unnecessary investigations, medications or referrals are unethical.

### Ethics in research

The whole world has become very vigilant on the question of research involving human subjects. There is large number of international and national guidelines for research involving human subjects. The Indian Council of Medical Research has published such guideline for uses in India.[16] Some of the basic issues to be addressed in research are following:

The research project should be carefully planned so that it can answer the research questions that it wanted to answer. Poorly conceptualized research project is highly unethical as it wastes national resources and put unnecessary strain on the subjects.

The welfare of the patient should be prime concern. Research that could produce potential harm should be carefully monitored. The use of very complicated informed consent is often counter productive and the researchers obtain consent for what they want. The offer of money for travel etc and use of new imported medicines are the inducement used to obtain consent from poor unsuspecting patients.

Use of placebo in patients where standard treatments are available poses serious ethical dilemma. The institutional review boards (IRB) are mandatory for ethical clearance of research projects. There are no restrictions on who can select the members of IRB. Often the institutional heads appoint an IRB that is convenient to them. Most IRB do not pay enough emphasis on the follow up of research project.

## CONCLUSIONS

Ethical practice is fundamental to effective and rational treatment.

Ethical aspects of psychiatric practice have not been properly discussed and there are no effective guidelines to guide the psychiatrists in there clinical work.

Ethics are evolving and one should be ready to change with times.

There are no irrefutable principles and the clinician has to learn to find the most suitable and best fit for each clinical situation.

There is a need to evolve ethical guideline applicable to psychiatry for this country.

Till such time such guidelines are not available, the basic rule that should guide every clinician’s behavior is that one should not do to others that one would not like to be done to him.
